# Supplementary material for: Sensitivity of soil hydrogen uptake to natural and managed moisture dynamics in a semiarid urban ecosystem
Source: PeerJ. 2022 Mar 17;10:e12966. doi: 10.7717/peerj.12966 (PMC8934528; doi:10.7717/peerj.12966)
Supplement: Supplemental Information 1 — Cumulative Daily precipitation in mm, daily average temperature with the standard deviation. Blue, starred text notes sampling day. [file peerj-10-12966-s001.docx]

| Day of Year | Precipitation (mm) | Air Temperature (°C; Daily Average) | Air Temperature (°C; Standard Deviation) |
| --- | --- | --- | --- |
| 148 | 0 | 27.94 | 7.69 |
| 149 | 0 | 29.89 | 8.20 |
| 150 | 0 | 30.68 | 6.91 |
| 151 | 0 | 30.38 | 7.31 |
| 152 | 0 | 29.78 | 8.47 |
| 153 | 0 | 31.20 | 9.30 |
| 154 | 0 | 31.78 | 9.40 |
| 155 | 0 | 32.53 | 9.13 |
| 156 | 0 | 32.71 | 7.05 |
| 157 | 0 | 33.11 | 7.06 |
| 158* | 0 | 33.02 | 7.58 |
| 204 | 0 | 37.12 | 7.02 |
| 205 | 0 | 37.86 | 6.68 |
| 206 | 0 | 37.98 | 6.40 |
| 207 | 0 | 35.81 | 5.25 |
| 208 | 0 | 35.39 | 5.16 |
| 209 | 0 | 32.40 | 6.00 |
| 210 | 0 | 33.10 | 5.45 |
| 211 | 0 | 34.30 | 6.13 |
| 212 | 0 | 34.17 | 5.13 |
| 213 | 6.86 | 34.45 | 6.56 |
| 214* | 0 | 33.26 | 6.44 |
| 245 | 16.4 | 28.47 | 4.72 |
| 246 | 1.4 | 27.50 | 6.44 |
| 247 | 1.2 | 28.30 | 4.88 |
| 248 | 0 | 29.86 | 6.72 |
| 249 | 2 | 29.83 | 5.49 |
| 250 | 0 | 31.34 | 5.27 |
| 251 | 0 | 31.87 | 6.26 |
| 252 | 0 | 32.48 | 6.18 |
| 253 | 0 | 32.17 | 6.73 |
| 254 | 0 | 32.43 | 7.22 |
| 255* | 0 | 32.16 | 7.00 |
| 308 | 0 | 18.53 | 7.80 |
| 309 | 0 | 19.33 | 7.71 |
| 310 | 0 | 20.35 | 7.41 |
| 311 | 0 | 19.85 | 8.02 |
| 312 | 0 | 19.02 | 7.66 |
| 313 | 0 | 18.03 | 7.60 |
| 314 | 0 | 19.61 | 5.69 |
| 315 | 0 | 15.36 | 6.76 |
| 316 | 0 | 11.77 | 6.44 |
| 317 | 0 | 12.04 | 3.10 |
| 318* | 0 | 14.04 | 3.62 |
