# Supplementary material for: Sensitivity of soil hydrogen uptake to natural and managed moisture dynamics in a semiarid urban ecosystem
Source: PeerJ. 2022 Mar 17;10:e12966. doi: 10.7717/peerj.12966 (PMC8934528; doi:10.7717/peerj.12966)
Supplement: Supplemental Information 2 — Blue, starred text notes sampling day. [file peerj-10-12966-s002.docx]

| Day of Year | Treatment | Soil Moisture (Daily Mean) | Soil Temperature (Daily Mean) | Soil Moisture (Standard Deviation) | Soil Temperature (Standard Deviation) |
| --- | --- | --- | --- | --- | --- |
| 148 | Active | 0.14 | 27.7 | 0 | 1.01 |
| 148 | Control | 0.14 | 33.15 | 0.01 | 3.34 |
| 148 | Greywater | 0.14 | 25.27 | 0.01 | 0.28 |
| 148 | Passive | 0.12 | 28.41 | 0 | 1.06 |
| 149 | Active | 0.14 | 28.1 | 0 | 1.04 |
| 149 | Control | 0.14 | 33.68 | 0.01 | 3.35 |
| 149 | Greywater | 0.13 | 25.08 | 0 | 0.31 |
| 149 | Passive | 0.12 | 28.79 | 0 | 1.14 |
| 150 | Active | 0.14 | 28.53 | 0 | 0.94 |
| 150 | Control | 0.14 | 34.48 | 0.01 | 3.15 |
| 150 | Greywater | 0.13 | 25.45 | 0 | 0.27 |
| 150 | Passive | 0.12 | 29.32 | 0 | 1.03 |
| 151 | Active | 0.14 | 28.62 | 0 | 0.95 |
| 151 | Control | 0.14 | 34.5 | 0.01 | 3.15 |
| 151 | Greywater | 0.13 | 25.63 | 0 | 0.3 |
| 151 | Passive | 0.12 | 29.44 | 0 | 1.05 |
| 152 | Active | 0.13 | 28.65 | 0 | 1.07 |
| 152 | Control | 0.14 | 34.29 | 0.01 | 3.33 |
| 152 | Greywater | 0.13 | 25.67 | 0 | 0.26 |
| 152 | Passive | 0.12 | 29.5 | 0 | 1.14 |
| 153 | Active | 0.13 | 28.88 | 0 | 1.1 |
| 153 | Control | 0.14 | 34.61 | 0.01 | 3.46 |
| 153 | Greywater | 0.18 | 28.03 | 0.05 | 2.79 |
| 153 | Passive | 0.12 | 29.73 | 0 | 1.17 |
| 154 | Active | 0.13 | 29.24 | 0 | 1.14 |
| 154 | Control | 0.14 | 35.16 | 0.01 | 3.52 |
| 154 | Greywater | 0.18 | 28.08 | 0.02 | 0.64 |
| 154 | Passive | 0.12 | 30.08 | 0 | 1.2 |
| 155 | Active | 0.13 | 29.48 | 0 | 1.14 |
| 155 | Control | 0.14 | 35.57 | 0.01 | 3.47 |
| 155 | Greywater | 0.14 | 27.04 | 0.01 | 0.31 |
| 155 | Passive | 0.12 | 30.34 | 0 | 1.2 |
| 156 | Active | 0.13 | 29.85 | 0 | 1.04 |
| 156 | Control | 0.14 | 36.07 | 0.01 | 3.12 |
| 156 | Greywater | 0.14 | 27.06 | 0 | 0.32 |
| 156 | Passive | 0.12 | 30.77 | 0 | 1.13 |
| 157 | Active | 0.13 | 30.33 | 0 | 0.9 |
| 157 | Control | 0.14 | 36.47 | 0 | 2.97 |
| 157 | Greywater | 0.13 | 27.29 | 0 | 0.29 |
| 157 | Passive | 0.12 | 31.3 | 0 | 0.99 |
| 158* | Active | 0.13 | 30.27 | 0 | 0.9 |
| 158* | Control | 0.14 | 36.33 | 0.01 | 3.03 |
| 158* | Greywater | 0.14 | 27.69 | 0 | 0.29 |
| 158* | Passive | 0.12 | 31.32 | 0 | 1.01 |
| 204 | Active | 0.14 | 33.08 | 0 | 0.85 |
| 204 | Control | 0.18 | 39 | 0 | 2.83 |
| 204 | Greywater | 0.16 | 30.76 | 0.01 | 0.34 |
| 204 | Passive | 0.14 | 33.55 | 0 | 1.07 |
| 205 | Active | 0.14 | 33.15 | 0 | 0.62 |
| 205 | Control | 0.18 | 39.18 | 0 | 2.3 |
| 205 | Greywater | 0.14 | 30.68 | 0 | 0.25 |
| 205 | Passive | 0.14 | 33.77 | 0 | 0.76 |
| 206 | Active | 0.14 | 33.47 | 0 | 0.67 |
| 206 | Control | 0.18 | 39.63 | 0 | 2.63 |
| 206 | Greywater | 0.14 | 30.82 | 0 | 0.32 |
| 206 | Passive | 0.14 | 34.13 | 0 | 0.9 |
| 207 | Active | 0.14 | 33.88 | 0 | 0.44 |
| 207 | Control | 0.17 | 39.53 | 0 | 1.91 |
| 207 | Greywater | 0.17 | 31.4 | 0.02 | 0.21 |
| 207 | Passive | 0.14 | 34.44 | 0 | 0.6 |
| 208 | Active | 0.14 | 34 | 0 | 0.56 |
| 208 | Control | 0.17 | 39.58 | 0 | 2.55 |
| 208 | Greywater | 0.14 | 31.1 | 0 | 0.31 |
| 208 | Passive | 0.14 | 34.5 | 0 | 0.79 |
| 209 | Active | 0.14 | 34.05 | 0 | 0.47 |
| 209 | Control | 0.17 | 38.43 | 0 | 2.47 |
| 209 | Greywater | 0.14 | 30.95 | 0 | 0.28 |
| 209 | Passive | 0.14 | 34.44 | 0 | 0.69 |
| 210 | Active | 0.13 | 34.07 | 0 | 0.51 |
| 210 | Control | 0.17 | 38.46 | 0 | 2.39 |
| 210 | Greywater | 0.14 | 30.92 | 0 | 0.23 |
| 210 | Passive | 0.14 | 34.39 | 0 | 0.69 |
| 211 | Active | 0.13 | 34.31 | 0 | 0.59 |
| 211 | Control | 0.17 | 39 | 0 | 2.51 |
| 211 | Greywater | 0.16 | 31.15 | 0.03 | 0.47 |
| 211 | Passive | 0.14 | 34.6 | 0 | 0.8 |
| 212 | Active | 0.13 | 34.4 | 0 | 0.54 |
| 212 | Control | 0.16 | 39.04 | 0 | 2.41 |
| 212 | Greywater | 0.15 | 31.23 | 0.01 | 0.32 |
| 212 | Passive | 0.13 | 34.59 | 0 | 0.72 |
| 213 | Active | 0.15 | 34.45 | 0.05 | 0.54 |
| 213 | Control | 0.17 | 39.16 | 0.03 | 2.33 |
| 213 | Greywater | 0.14 | 31.37 | 0 | 0.31 |
| 213 | Passive | 0.13 | 34.78 | 0 | 0.79 |
| 214* | Active | 0.22 | 33.68 | 0.01 | 0.36 |
| 214* | Control | 0.22 | 35.53 | 0 | 2.71 |
| 214* | Greywater | 0.14 | 31.08 | 0 | 0.27 |
| 214* | Passive | 0.13 | 33.43 | 0 | 0.62 |
| 245 | Active | 0.2 | 30.61 | 0.07 | 0.42 |
| 245 | Control | 0.2 | 32.05 | 0 | 2.31 |
| 245 | Greywater | 0.15 | 29.47 | 0 | 0.19 |
| 245 | Passive | 0.18 | 29.33 | 0 | 0.41 |
| 246 | Active | 0.27 | 29.33 | 0.01 | 0.47 |
| 246 | Control | 0.24 | 29.95 | 0 | 3 |
| 246 | Greywater | 0.14 | 28.45 | 0 | 0.35 |
| 246 | Passive | 0.19 | 28.29 | 0.01 | 0.67 |
| 247 | Active | 0.23 | 29.41 | 0.01 | 0.4 |
| 247 | Control | 0.23 | 30.1 | 0 | 1.88 |
| 247 | Greywater | 0.14 | 28.28 | 0 | 0.23 |
| 247 | Passive | 0.19 | 28.23 | 0 | 0.5 |
| 248 | Active | 0.22 | 29.58 | 0 | 0.62 |
| 248 | Control | 0.22 | 30.45 | 0 | 3.03 |
| 248 | Greywater | 0.14 | 28.27 | 0 | 0.4 |
| 248 | Passive | 0.19 | 28.34 | 0 | 0.8 |
| 249 | Active | 0.2 | 29.88 | 0 | 0.53 |
| 249 | Control | 0.22 | 31.03 | 0 | 2.64 |
| 249 | Greywater | 0.14 | 28.5 | 0 | 0.3 |
| 249 | Passive | 0.18 | 28.56 | 0 | 0.61 |
| 250 | Active | 0.19 | 29.95 | 0 | 0.59 |
| 250 | Control | 0.21 | 30.98 | 0 | 3.09 |
| 250 | Greywater | 0.14 | 28.37 | 0 | 0.39 |
| 250 | Passive | 0.17 | 28.45 | 0 | 0.69 |
| 251 | Active | 0.18 | 30.2 | 0 | 0.6 |
| 251 | Control | 0.21 | 32 | 0 | 2.76 |
| 251 | Greywater | 0.19 | 29.21 | 0.05 | 0.79 |
| 251 | Passive | 0.16 | 28.7 | 0 | 0.68 |
| 252 | Active | 0.17 | 30.5 | 0 | 0.61 |
| 252 | Control | 0.2 | 32.6 | 0 | 2.79 |
| 252 | Greywater | 0.18 | 29.55 | 0.01 | 0.27 |
| 252 | Passive | 0.16 | 28.98 | 0 | 0.67 |
| 253 | Active | 0.16 | 30.68 | 0 | 0.6 |
| 253 | Control | 0.2 | 32.91 | 0 | 2.67 |
| 253 | Greywater | 0.16 | 29.29 | 0.01 | 0.3 |
| 253 | Passive | 0.16 | 29.07 | 0 | 0.64 |
| 254 | Active | 0.16 | 30.73 | 0 | 0.61 |
| 254 | Control | 0.19 | 33.18 | 0 | 2.79 |
| 254 | Greywater | 0.15 | 29.3 | 0 | 0.37 |
| 254 | Passive | 0.15 | 29.13 | 0 | 0.7 |
| 255* | Active | 0.15 | 30.63 | 0 | 0.59 |
| 255* | Control | 0.19 | 33.17 | 0 | 2.61 |
| 255* | Greywater | 0.14 | 29.27 | 0 | 0.37 |
| 255* | Passive | 0.15 | 29.06 | 0 | 0.6 |
| 308 | Active | 0.21 | 17.7 | 0 | 0.45 |
| 308 | Control | 0.2 | 16.42 | 0 | 1.92 |
| 308 | Greywater | 0.2 | 20.43 | 0.02 | 0.41 |
| 308 | Passive | 0.19 | 17.93 | 0 | 0.6 |
| 309 | Active | 0.2 | 17.88 | 0 | 0.37 |
| 309 | Control | 0.19 | 16.94 | 0 | 1.63 |
| 309 | Greywater | 0.19 | 20.25 | 0.01 | 0.28 |
| 309 | Passive | 0.19 | 18.08 | 0 | 0.53 |
| 310 | Active | 0.2 | 17.79 | 0 | 0.39 |
| 310 | Control | 0.19 | 17 | 0 | 1.79 |
| 310 | Greywater | 0.16 | 19.86 | 0.01 | 0.27 |
| 310 | Passive | 0.19 | 18.08 | 0 | 0.59 |
| 311 | Active | 0.2 | 17.76 | 0 | 0.38 |
| 311 | Control | 0.19 | 17.05 | 0 | 1.68 |
| 311 | Greywater | 0.15 | 19.79 | 0 | 0.28 |
| 311 | Passive | 0.19 | 18.03 | 0 | 0.53 |
| 312 | Active | 0.24 | 17.41 | 0.03 | 0.35 |
| 312 | Control | 0.19 | 16.58 | 0 | 1.68 |
| 312 | Greywater | 0.14 | 19.56 | 0 | 0.28 |
| 312 | Passive | 0.19 | 17.73 | 0 | 0.5 |
| 313 | Active | 0.23 | 17.13 | 0.01 | 0.31 |
| 313 | Control | 0.19 | 16.23 | 0 | 1.52 |
| 313 | Greywater | 0.18 | 20.02 | 0.05 | 0.65 |
| 313 | Passive | 0.18 | 17.45 | 0 | 0.46 |
| 314 | Active | 0.22 | 17.21 | 0 | 0.34 |
| 314 | Control | 0.19 | 16.59 | 0 | 1.63 |
| 314 | Greywater | 0.2 | 19.9 | 0.02 | 0.27 |
| 314 | Passive | 0.18 | 17.5 | 0 | 0.49 |
| 315 | Active | 0.21 | 16.73 | 0 | 0.32 |
| 315 | Control | 0.19 | 15.41 | 0 | 1.38 |
| 315 | Greywater | 0.17 | 19.14 | 0.01 | 0.33 |
| 315 | Passive | 0.18 | 16.98 | 0 | 0.4 |
| 316 | Active | 0.2 | 15.73 | 0 | 0.38 |
| 316 | Control | 0.18 | 13.35 | 0 | 1.4 |
| 316 | Greywater | 0.18 | 18.19 | 0.03 | 0.37 |
| 316 | Passive | 0.18 | 15.87 | 0 | 0.46 |
| 317 | Active | 0.2 | 14.92 | 0 | 0.23 |
| 317 | Control | 0.18 | 12.57 | 0 | 1.22 |
| 317 | Greywater | 0.18 | 16.74 | 0.01 | 0.77 |
| 317 | Passive | 0.18 | 14.87 | 0 | 0.33 |
| 318* | Active | 0.2 | 14.68 | 0 | 0.36 |
| 318* | Control | 0.18 | 12.27 | 0 | 1.43 |
| 318* | Greywater | 0.16 | 15.76 | 0.01 | 0.16 |
| 318* | Passive | 0.18 | 14.55 | 0 | 0.39 |
