# Supplementary material for: Sensitivity of soil hydrogen uptake to natural and managed moisture dynamics in a semiarid urban ecosystem
Source: PeerJ. 2022 Mar 17;10:e12966. doi: 10.7717/peerj.12966 (PMC8934528; doi:10.7717/peerj.12966)
Supplement: Supplemental Information 3 — Pairwise comparisons using Tukey’s HSD (honest significant difference) with Bonferroni correction. [file peerj-10-12966-s003.docx]

| Season | term | group1 | group2 | estimate | conf.low | conf.high | p.adj | p.adj.signif |
| --- | --- | --- | --- | --- | --- | --- | --- | --- |
| Premonsoon | Treatment | Active | Control | 0.497 | -0.630 | 1.625 | 0.574 | ns |
| Premonsoon | Treatment | Active | Greywater | -0.878 | -2.005 | 0.250 | 0.150 | ns |
| Premonsoon | Treatment | Active | Passive | 0.480 | -0.647 | 1.607 | 0.601 | ns |
| Premonsoon | Treatment | Control | Greywater | -1.375 | -2.502 | -0.248 | 0.016 | * |
| Premonsoon | Treatment | Control | Passive | -0.017 | -1.145 | 1.110 | 1.000 | ns |
| Premonsoon | Treatment | Greywater | Passive | 1.358 | 0.230 | 2.485 | 0.017 | * |
| Midmonsoon | Treatment | Active | Control | -0.550 | -1.527 | 0.427 | 0.379 | ns |
| Midmonsoon | Treatment | Active | Greywater | -0.530 | -1.507 | 0.447 | 0.409 | ns |
| Midmonsoon | Treatment | Active | Passive | -0.315 | -1.292 | 0.662 | 0.775 | ns |
| Midmonsoon | Treatment | Control | Greywater | 0.020 | -0.957 | 0.997 | 1.000 | ns |
| Midmonsoon | Treatment | Control | Passive | 0.235 | -0.742 | 1.212 | 0.889 | ns |
| Midmonsoon | Treatment | Greywater | Passive | 0.215 | -0.762 | 1.192 | 0.912 | ns |
| Latemonsoon | Treatment | Active | Control | -0.080 | -0.962 | 0.802 | 0.993 | ns |
| Latemonsoon | Treatment | Active | Greywater | -0.083 | -0.965 | 0.800 | 0.992 | ns |
| Latemonsoon | Treatment | Active | Passive | 0.748 | -0.135 | 1.630 | 0.108 | ns |
| Latemonsoon | Treatment | Control | Greywater | -0.003 | -0.885 | 0.880 | 1.000 | ns |
| Latemonsoon | Treatment | Control | Passive | 0.828 | -0.055 | 1.710 | 0.069 | ns |
| Latemonsoon | Treatment | Greywater | Passive | 0.830 | -0.052 | 1.712 | 0.068 | ns |
| Postmonsoon | Treatment | Active | Control | 0.040 | -0.696 | 0.776 | 0.998 | ns |
| Postmonsoon | Treatment | Active | Greywater | 0.080 | -0.656 | 0.816 | 0.988 | ns |
| Postmonsoon | Treatment | Active | Passive | 0.025 | -0.711 | 0.761 | 1.000 | ns |
| Postmonsoon | Treatment | Control | Greywater | 0.040 | -0.696 | 0.776 | 0.998 | ns |
| Postmonsoon | Treatment | Control | Passive | -0.015 | -0.751 | 0.721 | 1.000 | ns |
| Postmonsoon | Treatment | Greywater | Passive | -0.055 | -0.791 | 0.681 | 0.996 | ns |
