# Supplementary material for: Sensitivity of soil hydrogen uptake to natural and managed moisture dynamics in a semiarid urban ecosystem
Source: PeerJ. 2022 Mar 17;10:e12966. doi: 10.7717/peerj.12966 (PMC8934528; doi:10.7717/peerj.12966)
Supplement: Supplemental Information 4 — Pairwise comparisons using Dunn Test with Bonferroni correction. [file peerj-10-12966-s004.docx]

| term | group1 | group2 | n1 | n2 | statistic | p | p.adj |
| --- | --- | --- | --- | --- | --- | --- | --- |
| Hydrogen.flux | Dry (2% GWC) | Moist (10 GWC) | 16 | 16 | -1.932 | 0.053 | 0.160 |
| Hydrogen.flux | Dry (2% GWC) | Wet (20% GWC) | 16 | 16 | -5.758 | 0.000 | 0.000 |
| Hydrogen.flux | Moist (10% GWC) | Wet (20% GWC) | 16 | 16 | -3.826 | 0.000 | 0.000 |
